# Modulation of Oxidative Status by Normoxia and Hypoxia on Cultures of Human Dermal Fibroblasts: How Does It Affect Cell Aging?

**DOI:** 10.1155/2018/5469159

**Published:** 2018-09-23

**Authors:** Elisabetta Damiani, Francesca Brugè, Ilenia Cirilli, Fabio Marcheggiani, Fabiola Olivieri, Tatiana Armeni, Laura Cianfruglia, Angelica Giuliani, Patrick Orlando, Luca Tiano

**Affiliations:** ^1^Dipartimento di Scienze della Vita e dell'Ambiente, Università Politecnica delle Marche, Ancona, Italy; ^2^Dipartimento di Scienze Cliniche, Specialistiche ed Odontostomatologiche, Università Politecnica delle Marche, Ancona, Italy; ^3^Dipartimento di Scienze Cliniche e Molecolari, Università Politecnica delle Marche, Ancona, Italy; ^4^Center of Clinical Pathology and Innovative Therapy, IRCCS INRCA National Institute, Ancona, Italy

## Abstract

Reactive oxygen species (ROS) production in the skin is among the highest compared to other organs, and a clear correlation exists between ROS production and skin aging. Many attempts are underway to reduce oxidative stress in the skin by topical treatment or supplementation with antioxidants/cosmeceuticals, and cultures of human dermal fibroblasts (HDF) are widely used for these studies. Here, we examined the influence of oxygen tension on cell aging in HDF and how this impacted ROS production, the enzymatic and nonenzymatic antioxidant response system, and the efficacy of this defense system in limiting DNA damage and in modulating gene expression of proteins involved in the extracellular matrix, linked to skin aging. We investigated a selection of parameters that represent and reflect the behavior of cellular responses to aging and oxygen tension. Serial passaging of HDF under normoxia (21%) and hypoxia (5%) leads to cell aging as confirmed by *β*-galactosidase activity, p16 expression, and proliferation rate. However, in HDF under 21% O_2_, markers of aging were significantly increased compared to those under 5% O_2_ at matched cell passages despite having lower levels of intracellular ROS and higher levels of CoQ_10_, total GSH, SOD1, SOD3, and mitochondrial superoxide anion. miRNA-181a, which is known to be upregulated in HDF senescence, was also analyzed, and indeed, its expression was significantly increased in old cells at 21% O_2_ compared to those at 5% O_2_. Upregulation of MMP1 and downregulation of COL1A1 along with increased DNA damage were also observed under 21% O_2_ vs 5% O_2_. The data highlight that chronic exposure to atmospheric 21% O_2_ is able to trigger hormetic adaptive responses in HDF that however fail, in the long term, to prevent cellular aging. This information could be useful in further investigating molecular mechanisms involved in adaptation of skin fibroblasts to oxidative stress and may provide useful hints in addressing antiaging strategies.

## 1. Introduction

Oxygen is essential for any form of aerobic life, and at normal oxygen levels, cellular functions are maintained in a physiological state. However, one of the consequences of oxygen metabolism in cells is the generation of potentially harmful reactive oxygen species (ROS) in nearly all intracellular organelles and compartments [1]. These are physiological products of aerobic life, and to cope with them, cells have developed different strategies for converting them into less toxic species thus preventing their accumulation. In fact, the cumulative result of oxidative damage to cells due to increased ROS production and/or decreased antioxidant defenses is associated with the aging process [2, 3]. There is probably no better organ in the human body than the skin where this is most evident. The load of ROS in this organ is among the highest compared to other organs, and in many cases, a clear correlation between ROS originating from external and internal insults and a proaging effect can be found [4]. Furthermore, extrinsic aging in this organ due to lifestyle factors like smoking and environmental factors such as sun exposure and pollution that accelerate natural aging is at least as important as intrinsic aging, which is mainly due to genetic background [5]. A combination of all these factors leads to ROS generation and oxidative stress, which in turn causes cellular damage, disruption of the redox status, and loss of molecular functions in skin cells.

The most direct and visible signs of aging skin are the appearance of wrinkles, fine lines, sagging, thinning, dryness, and irregular skin pigmentation [5, 6]. Hence, the skin represents a useful model for studying aging in humans because it is readily visible and accessible. Moreover, because of the continual rise in the aging population, there is increasing interest in the study and use of antiaging compounds, whether of natural or synthetic origin, that can improve both the health and appearance of the skin [7, 8]. Since an increase in ROS levels over time is a feature of the skin, many attempts are being made to quench these ROS by topical treatment or supplementation with antioxidants in the hope to improve the aged skin [9]. Part of this quest involves *in vivo* and *in vitro* testing methods, and among the latter, cultures of human dermal fibroblasts (HDF) are widely used to provide important insights into intrinsic and extrinsic skin aging [10, 11]. Skin cells play a central role in skin aging studies, since they are responsible for synthesizing extracellular matrix proteins such as collagen and elastin, important for maintaining healthy connective tissue and therefore skin integrity.

Cell culture studies are commonly performed under standard atmospheric oxygen tension, ~140 mm Hg (21% O_2_), which is far from the physiological oxygen tension experienced by HDF which in normal skin is at 37.5–50 mm Hg (~6% O_2_) [12]. These conditions represent per se a mild oxidative stress that has been shown to accelerate the development of a senescent phenotype. In fact, under atmospheric oxygen tension (21% O_2_), HDF rapidly switch from a mitotic to a postmitotic phenotype, whereas under lower oxygen tension (4%), this induction is largely prevented [13].

Based on the above statement, in the current study, we decided to first explore how atmospheric oxygen tension (21% O_2_) could impact aging of HDF compared to those cultured under lower oxygen tension (5% O_2_). For this purpose, we determined the levels of commonly used biological markers of aging (*β*-galactosidase activity, p16 gene expression, and proliferation rate) at different serial passages. Once this was established, we investigated whether there were differences in the oxidative status of HDF since they were subjected to normoxic (21% O_2_) and hypoxic (5% O_2_) conditions, by measuring specific molecular and biochemical parameters. These included production of intracellular and mitochondrial ROS, antioxidant defense system responses, and DNA damage. In addition, we also investigated if the different oxygen tensions experienced by HDF could impact the mRNA expression levels of extracellular matrix (ECM) modeling proteins, important in dermal aging, namely, matrix metalloproteinase 1 (MMP1) and collagenase I (COL1A1). Finally, in order to describe the molecular mechanisms underlying altered gene expression and senescent phenotype, we also quantified the relative expression of microRNA-181a. MicroRNAs (miRNAs or miRs) are small noncoding RNA that can be produced in a regulated manner and act as controllers of protein expression modulating multiple functions including proliferation and differentiation in aging (senescence-associated miRNA (SA-miR)), inflammation (inflamma-miR), and cell death [14]. Notably, a limited number of miRNAs can be found associated with mitochondria (mito-miR) [15]. Mito-miR roles are still largely unknown, but one of the hypotheses is that they may modulate the expression of mitochondrial functional proteins [16]. miR-181a was chosen for analysis since it belongs to a subset of miRNA involved in senescence and inflammation and it directly affects mitochondrial function [17].

## 2. Materials and Methods

### 2.1. Cell Culture

Primary cultures of human dermal fibroblasts (HDF) were purchased from Istituto Zooprofilattico Sperimentale, Brescia, Italy, and cultured in Minimum Essential Medium (MEM) (GIBCO) supplemented with 10% SERA Plus specially processed fetal bovine serum (FBS) (PAN biotech GmbH), penicillin (100 U/mL), streptomycin (100 *μ*g/mL), and L-glutamine (2 mM). Upon thawing, a batch of cells was divided into two 25 cm^2^ flasks for two distinct culture conditions—one for cultures under standard atmospheric oxygen tension (21% O_2_) using an Heraeus BB15 incubator (Thermo Scientific, Germany) and the other for cultures under low oxygen tension (5% O_2_) using an HERAcell 150i incubator (Thermo Scientific, Germany) equipped with an oxygen sensor and nitrogen supply. Both incubators were maintained at 37°C and 5% CO_2_ under a humidified atmosphere. For the experiments, cells grown under the two oxygen tensions were seeded at an optimal density of 10 × 10^3^ cell/cm^2^. Cell culture medium was changed every 2-3 days and fibroblasts were passaged at 80% confluence by trypsinization. Cells were serially cultured under their respective oxygen tension and expanded until enough cells were obtained for all experiments. For the experiments, the cells considered as young (Y) were those collected at the 10th passage (p) whereas those considered as old (O) were collected at the 24th p. The cells were always subcultured to maintain equivalent passage numbers.

### 2.2. Growth Curve Determination and Cellular Proliferation Assay

Cell growth was determined in young cells cultured under two different oxygen tensions: 5% and 21% O_2_, by counting cell numbers in a haemocytometer, in the linear range of growth rate (24–48–72 h). The viability of the cells was estimated by examining their ability to exclude Trypan blue (0.1% in 0.9% NaCl). The cell population doubling time was calculated on data extrapolated from their growth curves using the formula (*t*2 − *t*1)^∗^ln 2/ln (final concentration/initial concentration), to obtain a doubling time in relation to their time in culture. For cellular proliferation assay, the fluorescence dye, carboxyfluorescein diacetate succinimidyl ester (CFSE) (Merck Millipore), was used which is able to directly monitor divided cells by flow cytometry. This dye diffuses freely into cells and is retained within the cell without affecting cellular function [18]. For each round of cell division, the relative fluorescence intensity of the dye decreases by half. Briefly, cells seeded on 6-well plates were washed and incubated with 2.5 *μ*M CFSE in PBS for 15 min at 37°C. After medium replacement, cells were collected at different times (days 0, 3, 6, 8, and 10) and propidium iodide (PI) (2.5 ng/*μ*L) was added just before cytometric evaluation to discriminate and exclude dead cells from the analysis. The analyses were conducted on a Guava EasyCyte flow cytometer (Merck Millipore) using an excitation wavelength of 488 nm and the following gain settings: FSC: 20.7; SSC: 9.93; RED: 7.03; GRN: 1; and threshold: 1000. The results were analyzed using InCyte software which was the software used in all subsequent experiments using the Guava EasyCyte flow cytometer. The proliferation index was calculated from the cell fluorescence distribution which represents the ratio between the medians of the CFSE fluorescence distribution at each time point and time 0. This indicates how many times the fluorescence intensity has been diluted at a specific time point; therefore, it is related to cellular division and proliferation.

### 2.3. Senescence-Associated *β*-Galactosidase Staining

Senescence-associated *β*-galactosidase activity (SA-*β*-gal), a common molecular marker of HDF cellular aging *in vitro* [19], was determined using a Senescence Detection Kit (BioVision, USA) according to the manufacturer's instructions. The development of blue color, which indicates SA-*β*-gal activity, was visible after overnight incubation at 37°C with the staining solution mix at pH 6. The percentage of blue cells observed in 100 cells under a standard light microscope was then calculated.

### 2.4. Intracellular ROS Assay

As indicator of intracellular ROS formation, the leuco dye carboxy-2,7-dichlorofluorescein diacetate (carboxy-H_2_DCFDA) (Invitrogen) was employed as described elsewhere [20]. Briefly, after washing the cells, a solution of carboxy-H_2_DCFDA 10 *μ*M in PBS was added to each sample and cells were incubated in the dark for 30 min at 37°C. After trypsinization, cells were harvested and centrifuged and the cell pellet was resuspended in approximately 100 *μ*L of PBS. An aliquot of 30 *μ*L from each sample was then added to 270 *μ*L of a solution of Guava ViaCount (Merck Millipore). This is a fluorescent stain formulation which provides sensitive, accurate detection of viable, apoptotic, and dead cells in flow cytometry. The analyses for cell viability and intracellular ROS production were then conducted simultaneously on the Guava EasyCyte flow cytometer using an excitation wavelength of 488 nm. Emissions were recorded using the green channel for carboxy-DCF and the red and yellow channels for the ViaCount dye, using the following gain settings: FSC 20.7; SSC 9.93; GRN 3.51; YEL 15.3; RED 11.8; and a threshold of 1000 on FSC. The fluorescence intensity was recorded on an average of 5000 cells from each sample. Counterstaining with ViaCount was necessary in order to evaluate intracellular levels of ROS only in viable cells. In fact, exclusion of cells with compromised cell membrane integrity is essential in order to avoid false negatives due to loss of carboxy-H_2_DCFDA from permeable cells.

### 2.5. Mitochondrial Superoxide Anion Assay

For monitoring mitochondrial superoxide anion generation, we used the FlowCellect™ MitoStress Kit (Merck Millipore) which allows the measurement of superoxide anion by the membrane-permeant dye MitoSOX Red. The cell staining protocol was followed as reported in the kit's manual for cultured adherent cells. In particular, we used 10 × 10^5^ cells/sample. The analyses were conducted on the Guava EasyCyte flow cytometer using an excitation wavelength of 488 nm. Emissions were recorded using the red channel for MitoSOX Red with the following gain settings: FSC 20.7; SSC 9.93; YEL 19.9; and a threshold of 1000 on FSC. The fluorescence intensity was recorded on an average of 5000 cells from each sample. Specifically, for data analysis, two regions relative to yellow fluorescence, proportional to mitochondrial superoxide production, were arbitrarily defined. These were based on preliminary experiments on cells exposed to rotenone representing the positive control. Specifically, rotenone at a concentration of 0.6 *μ*M for 3 days induces fibroblast senescence [21] and leads to an increase in intracellular superoxide anion. Based on the fluorescence distribution between nontreated and treated cells with rotenone, two gates were arbitrarily set to define the two regions. These settings were then kept for all subsequent experiments for cells at different passages under the two oxygen tensions, and the relative percentage of cells in each region was calculated.

### 2.6. Determination of Coenzyme Q_10_

Coenzyme Q_10_ (CoQ_10_) levels and its oxidative status were assayed in fibroblasts grown on 6-well plates using a dedicated high-performance liquid chromatography (HPLC) system with an electrochemical detector capable of detecting both reduced and oxidized forms (ECD; Shiseido, Tokyo, Japan) as reported in [22]. CoQ_10_ concentration was verified by using a single dilution step. Briefly, after cell harvesting, the cell pellet was resuspended in 50 *μ*L PBS and extracted with 250 *μ*L propanol. After vigorous vortexing, the extraction mixture was centrifuged for 1 min at 13,000g at 4°C, and 40 *μ*L of supernatant was injected into the HPLC system. Intracellular total CoQ_10_ levels are expressed as *μ*g/mg of protein, while the oxidative status of CoQ_10_ is reported as the percentage of oxidized CoQ_10_/total CoQ_10_. The protein concentration was determined using the Bradford assay.

### 2.7. Determination of Total Glutathione

Total glutathione (GSH + GSSG) was measured spectrophotometrically using the glutathione reductase (GR) recycling assay in the presence of 5,5′-dithiobis(2-nitrobenzoic) acid (DTNB) [23]. Briefly, the absorbance at 412 nm generated after reaction with DTNB was measured and then total glutathione concentration was calculated using a calibration curve obtained with known concentrations of GSH. Before analysis, cells were trypsinized, washed twice in cold PBS, and quickly centrifuged. For total glutathione determination, the pellet was resuspended with 1% sulphosalicylic acid, vortexed, and then incubated for 30 min at 4°C. The samples were centrifuged for 2 min at 2300g and total glutathione was quantified on the supernatant. Finally, the pellet was resuspended with 1 M NaOH for recovery and quantification of proteins by the Bradford method using BSA as standard. Intracellular total glutathione is expressed as nmol/mg of protein.

### 2.8. Total RNA Extraction and cDNA Synthesis

Total RNA was isolated from fibroblasts grown under the two oxygen tensions (21% O_2_ and 5% O_2_), using the NucleoSpin RNA kit (MACHEREY-NAGEL) according to the manufacturer's instructions. The RNA purity and concentration were measured on a NanoDrop spectrophotometer. Approximately 400 ng of RNA from each sample was converted to cDNA using the iScript™ cDNA synthesis kit (Bio-Rad) according to the manufacturer's instructions.

### 2.9. Quantitative Real-Time PCR (qPCR)

qPCR reactions were conducted on a MyiQ Single-Color Real-Time PCR Detection System (Bio-Rad) in a 15 *μ*L total reaction volume, using the iQ™ SYBR Green supermix (Bio-Rad). The primer sequences for the genes of interest, p16, CAT, SOD1, SOD3, MMP1, and COL1A1, are reported in the supplementary data (Supplementary Table S1). Since no gene expression analysis using RT-qPCR (reverse transcription quantitative real-time PCR) can be reliable without first choosing the appropriate, endogenous control genes for relative quantification under the experimental conditions used, a geNorm analysis was initially performed [24]. For this purpose, a panel of six potential reference genes that are constitutively expressed and that span a range of ubiquitous and different cellular functions was chosen.

All primers were used at a concentration of 400 nM, except for *β*-actin that was used at a concentration of 300 nM. Each reaction was run in duplicate, and for each gene, a no-template control was included. The qPCR was programmed to start with a 3 min denaturation step at 95°C for polymerase activation, followed by 40 cycles of 15 sec denaturation at 95°C and 30 sec of annealing/extension at 60°C, during which fluorescence was measured. Next, a melting curve was constructed by increasing the temperature from 55 to 95°C in sequential steps of 0.5°C for 6 sec while continuously monitoring fluorescence. All PCR efficiencies were between 90 and 110%. For each sample, at least three biological replicates were performed. The mRNA expression of the genes of interest was calculated according to the delta-delta Ct method (2^−ΔΔCt^) using the three reference genes GAPDH, *β*-actin, and SDHA for normalization. The results obtained were analyzed using the iQ5 Software (Bio-Rad), and the mean of the normalized expression values obtained from three independent experiments was calculated for data analysis.

### 2.10. miRNA 181a Expression

For miRNA analysis, total RNA from HDF was isolated using the Norgen Biotek Kit (#37500, Thorold, ON, Canada), according to the manufacturer's protocol, and the RNA was stored at −80°C until use. miRNA expression was quantified by quantitative real-time PCR (RT-qPCR) using TaqMan miRNA assay (catalog #4427012—Thermo Fisher Scientific), according to the manufacturer's instructions. Briefly, miRNA was reverse transcribed using TaqMan miRNA RT kit (4366596—Thermo Fisher Scientific), using a miR-specific stem-loop primer. RT mix was prepared as follows: 3.34 *μ*L of input RNA, 2 *μ*L of each miR-specific stem-loop primer, 1 *μ*L of 10 mM dNTPs, 0.67 *μ*L of reverse transcriptase, 1.26 *μ*L of RNase inhibitor (dil 1 : 10), 1 *μ*L of 10x buffer, and 0.73 *μ*L of H_2_O. The RT mix was incubated for 30 min at 16°C, for 30 min at 42°C and for 5 min at 85°C.

Subsequently, a reaction mixture was prepared containing 5 *μ*L 2x TaqMan Universal Master mix no UNG (4440040—Thermo Fisher Scientific), 0.5 *μ*L 20x TaqMan MicroRNA Assay, containing PCR primers and probes (5'-FAM), and 2.66 *μ*L RT product. Amplification consisted of an initial step at 95°C for 2 min, followed by 40 cycles of 95°C for 15 sec and 60°C for 1 min. Data were analyzed with Rotor-Gene Q (QIAGEN, Hilden, Germany), and the RT-qPCR data were standardized to RNU44. The 2^−ΔΔCT^ method was used to determine miRNA expression.

### 2.11. Comet Assay

For the comet assay which reveals single- and double-strand DNA damage, HDF were detached and counted using a solution of Guava ViaCount on the Guava EasyCyte flow cytometer. Aliquots containing 10,000 cells from each sample were collected, and a freezing solution containing PPS albumin 5% (60%), albumin 20% (20%), and DMSO (20%) was added to this cell suspension in a 1 : 1 ratio. After mixing, the solution was kept at −80°C until analysis. Upon analysis, samples were transferred on ice and a thawing solution containing albumin 20% (2.5%), dextran (50%), and PBS was added. A volume corresponding to 4000 cells was transferred to Eppendorf tubes and centrifuged for 10 min at 800g at 4°C. The supernatant was removed, and cells were resuspended in 80 *μ*L of 0.7% low-melting-point agarose from which 35 *μ*L were taken and placed on precoated, high-throughput, comet assay slides (Trevigen, Gaithersburg, MD) for technical duplicates. The microgels on the slides were then allowed to solidify on ice for 15 min at 4°C. Subsequently, the slides were immersed for 2 h at 4°C in the dark, in ice-cold, freshly prepared lysis solution (2.5 M NaCl, 100 mM Na_2_EDTA, 10 mM Tris-HCl, 1% Triton X-100, and 10% DMSO, adjusted to pH 10). This was followed by DNA unwinding in freshly prepared alkaline buffer (1 mM Na_2_EDTA, pH 13) for 30 min at 4°C. Electrophoresis was then performed for 20 min at 1 V/cm in the same buffer. After washing in H_2_O, neutralization in Tris buffer (pH 7.5) for 5 min, and dehydration in 50% methanol, the slides were dried at 50°C. DNA on each slide was stained with 15 *μ*L ethidium bromide (20 *μ*g/mL), and the comets were analyzed using fluorescence microscopy using a dedicated imaging software developed as previously reported [25]. For each comet, data relative to tail length (TL), tail migration (TMi), percent tail DNA (TI), and tail moment (TM) were recorded. The results are reported as a % of TI.

### 2.12. Statistical Analysis

The data are the average of at least three independent experiments, and for the descriptive statistics, mean ± standard error of the mean (SEM) was used for measurements expressed as percentage, whereas all other measurements are reported as mean ± standard deviation (SD). Absence of normal distribution of the data was verified using the Shapiro-Wilk test, and consequently, significance of differences was estimated using the nonparametric *t*-test. The statistical analysis was performed using GraphPad Prism 7. The significance level was set at *p* ≤ 0.05 (^∗^), *p* < 0.01 (^∗∗^), or *p* < 0.001 (^∗∗∗^).

## 3. Results

### 3.1. Cellular Proliferation Decreases with Cell Passages and Increases with Oxygen Tension

**Figure 1:**
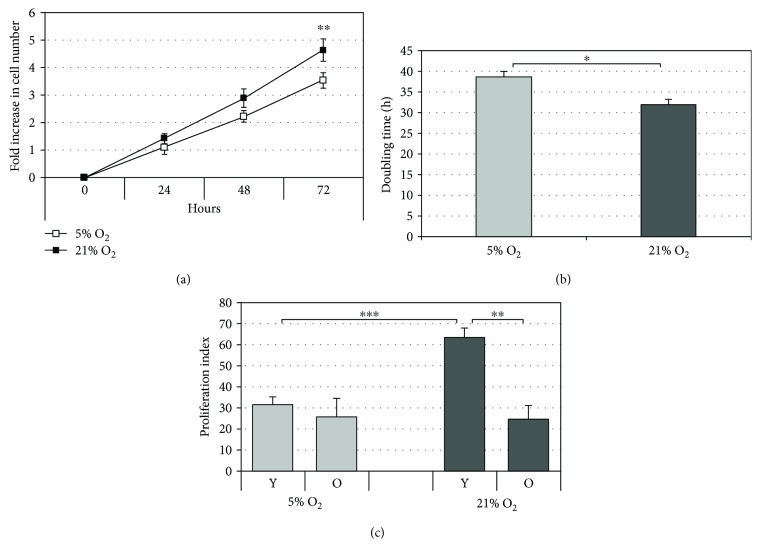
Cellular proliferation analysis in HDF under 5% O_2_ and 21% O_2_ culture conditions. (a) Growth curve of young HDF cells cultured under 5% O_2_ (grey bar) and 21% O_2_ (black bar). The points correspond to 24–48–72 h of culture and indicate fold increase in cell number. Mean values were calculated on at least six replicates. (b) The doubling time of young HDF grown under the two different oxygen tensions (*n* = 6). (c) Proliferation index of the cells cultured under 5% and 21% O_2_ calculated between day 0 and day 3 after CFSE staining in both young (Y) and old (O) cells. Error bars represent ± SD. ^∗^ *p* < 0.05; ^∗∗^ *p* < 0.01; ^∗∗∗^ *p* < 0.001.

To investigate the effects of oxygen concentration on the proliferative capacity of HDF during aging, multiple assays were performed. Initially, to elucidate the effect of oxygen on the proliferation capacity of HDF, the number of young cells grown in either 21% or 5% O_2_ tension was monitored for 72 h and the standard growth curve was generated. The recorded cell counts demonstrated increased cell proliferation under 21% O_2_ compared to 5% O_2_ with a major significant gap after 72 h (*p* < 0.01) (Figure 1(a)). The doubling time of the cells calculated from the data extrapolated from the growth curve showed a greater doubling time for the cells grown under 5% O_2_ relative to those grown under 21% oxygen (*p* < 0.05) (Figure 1(b)). Finally, the proliferation index of young and old cells grown under the two oxygen tensions was also investigated using the specific dye (CFSE). The results (Figure 1(c)) show that upon increasing cell passages, regardless of whether cells were cultured under 21% or 5% O_2_ tension, there is a decrease in the proliferation index which is more evident under 21% O_2_. It is noteworthy that this remarkable difference is due to a greater proliferative capacity of young cells cultured at 21% O_2_ (higher proliferation index) compared to passage-matched cells grown under 5% O_2_. Figure 1(c) shows the results obtained between day 0 and day 3 after probe (CFSE) staining when the proliferation rate of cells under the two culture conditions was remarkably different.

### 3.2. Markers of Senescence Increase with Cell Passages

**Figure 2:**
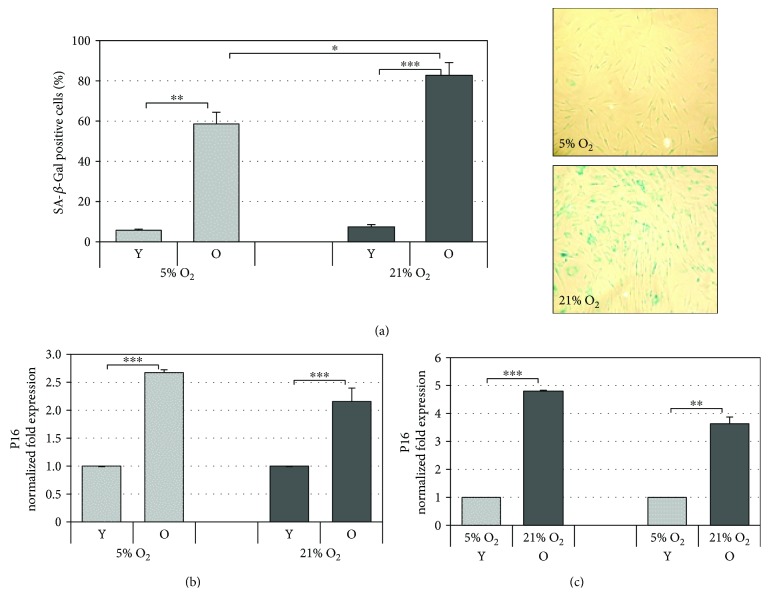
Markers of cellular aging. (a) Quantitative analysis of positive *β*-galactosidase-stained cells in HDF during cellular aging under 5% and 21% O_2_ culture conditions. The percentage of blue cells observed in old cells (right panel) under a standard light microscope was calculated. Error bars represent ± SEM. (b) p16 mRNA modulation during aging in old (O) HDF with respect to young (Y) ones cultured under normoxic (21% O_2_) and hypoxic (5% O_2_) conditions; (c) p16 mRNA expression in cells cultured under 21% O_2_ with respect to 5% O_2_ (right panel). The gene was normalized vs GAPDH/*β*-actin/SDHA, and the results are reported as normalized fold expression. Error bars represent ± SD ^∗^ *p* < 0.05; ^∗∗^ *p* < 0.01; ^∗∗∗^ *p* < 0.001.

In order to evaluate the effect of different oxygen tensions on the aging progression, two markers of senescence were used in young and old cells cultured under 21% or 5% oxygen tension. Lysosomal senescence-associated enzyme SA-*β*-galactosidase is commonly used as a biomarker of aging because senescent cells show an increase in its activity. Therefore, HDF from young and old cells were compared in the context of SA-*β*-gal activity, under either 21% or 5% oxygen tension culture conditions, and the quantitative results are given in Figure 2(a). As reported in the histogram, SA-*β*-gal-positive cells significantly increase under both culture conditions in old cells, indicative of cell aging. The percentage of blue cells is significantly higher in old fibroblasts grown under 21% O_2_ compared to those grown under 5% O_2_ (82.5 ± 4.1% vs 58.4 ± 6.5%; *p* < 0.05).

The p16 protein is the second marker of senescence that was considered because it appears to be more expressed in most senescent cells including fibroblasts and is known to increase with aging in several rodent and human tissues [26]. p16 gene expression was analyzed in cells cultured in parallel under both 21% O_2_ and 5% O_2_. The results reported in Figure 2(b) show, as expected, that upon increasing cell passages, regardless of whether cells were cultured under 21% or 5% O_2_, there is a significant increase in the expression levels of p16 mRNA in old cells with respect to young cells, and under 21% O_2_ with respect to 5% O_2_ in both young and old HDF (Figure 2(c)). These data further confirm, along with those of SA-*β*-gal, the aging of fibroblasts during serial passaging under both oxygen tensions and indicate that HDF cultured under 5% O_2_ are younger with respect to those cultured under 21% O_2_.

### 3.3. miRNA-181a Is Differently Regulated in Older Cells at Different Oxygen Tensions

**Figure 3:**
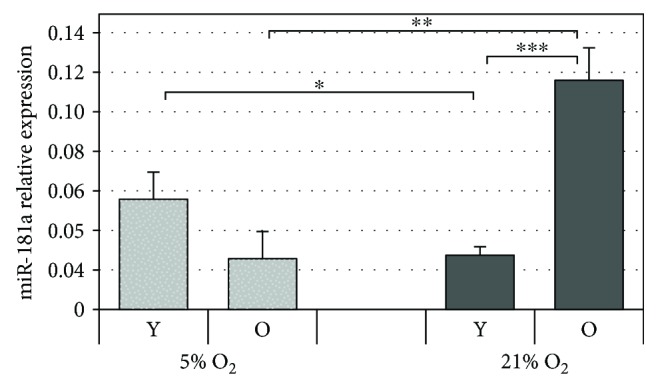
miRNA-181a expression. Modulation of miR-181a expression in young (Y) and old (O) HDF under 5% and 21% O_2_ culture conditions. The data were standardized to RNU44 and reported as relative expression with SD as error bars. ^∗^ *p* < 0.05.

miR-181a is a senescence-associated small noncoding RNA known to affect mitochondrial function by modulating mitochondrial transition pore. Specifically, miR-181a increase has been shown to be associated with decreased mitochondrial membrane potential and concomitant-enhanced susceptibility to apoptosis and ROS formation. Our results show that in comparing cells cultured under 21% O_2_ with those cultured under 5% O_2_, we observe downregulation of miR-181a in young cells while an opposite highly significant upregulation was observed in old cells (Figure 3). Taking into account the validated effect of miR-181a on mitochondrial function, this data helps to explain mitochondrial dysfunction experienced in old cells exposed to 21% O_2_. Moreover, it is known that miR-181a can affect glutathione peroxidase expression by downregulating it in conditions of oxidative stress [27]. This could further explain the different response of young and old cells where in the latter, modulation of antioxidant responses seems to be not as effective in counteracting oxidative insult.

### 3.4. Mitochondrial Superoxide Radical Increases in Older Cells and with Oxygen Tension

**Figure 4:**
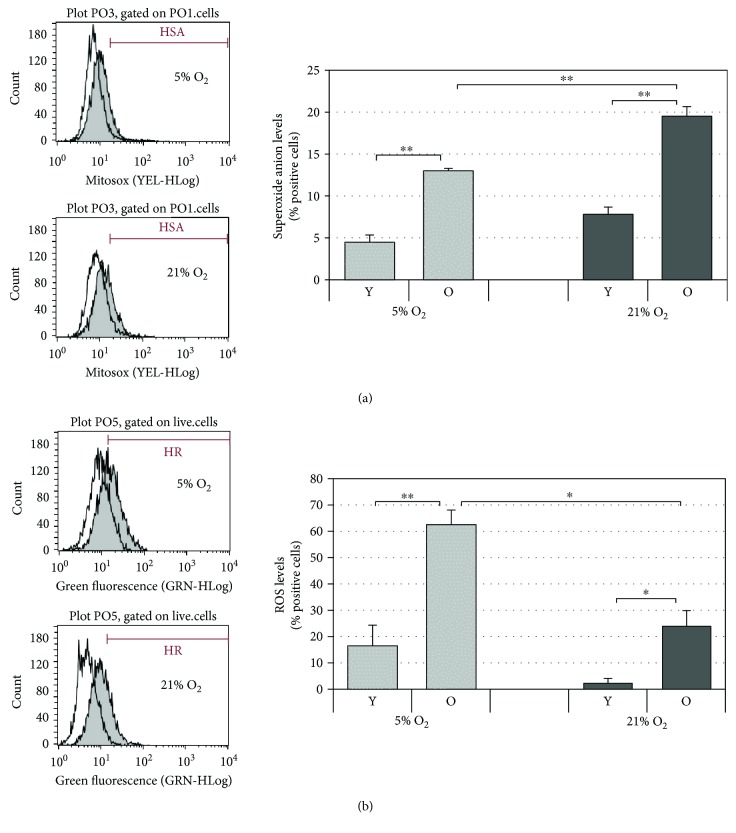
Superoxide anion level and ROS production in HDF during cellular aging under 5% O_2_ and 21% O_2_ culture conditions. (a) Flow cytometric analysis of mitochondrial superoxide anion production in young (Y) and old (O) HDF under 5% O_2_ and 21% O_2_ culture conditions determined using the MitoStress Kit. In the right panel, the superoxide anion level is reported as % of positive cells. (b) Flow cytometric analysis of intracellular levels of ROS in young (Y) and old (O) HDF under 5% O_2_ and 21% O_2_ determined using the carboxy-H_2_DCFDA assay. In the right panel, ROS production is reported as % of positive cells, obtained by subtraction of PI from carboxy-H_2_DCFDA fluorescence. In the left panel, representative cytometric histograms are reported for young cells (white) and old cells (grey) under the two oxygen tensions (21% and 5% O_2_). Error bars represent ± SEM. ^∗^ *p* < 0.05; ^∗∗^ *p* < 0.01.

The principal source of ROS in cells is mitochondria, and these are generated at the level of the electron transport chain. Electrons leaking from the electron transport chain directly reduce oxygen to the short-lived free radical superoxide anion (O_2_^•−^) which in turn generates other ROS such as hydrogen peroxide. Therefore, it was of interest to determine the levels of mitochondrial superoxide anion in HDF grown under the two different oxygen tensions. This was achieved by evaluating the percentage of cells showing an increase in MitoSOX Red fluorescence. The data reported in Figure 4(a) show that under both culture conditions, old cells have significantly higher levels of superoxide anion compared to young fibroblasts, in agreement with the mitochondrial free radical theory of aging [28] and with the markers of senescence reported above. Moreover, one can observe that the higher oxygen concentration (21% O_2_) leads to a greater percentage of cells with high levels of superoxide anion compared to those cultured under 5% O_2_. These differences are statistically different in old cells (*p* < 0.01).

### 3.5. Intracellular ROS Levels Increase in Older Cells but Not with Increased Oxygen Tension

Aging is known to be associated with increased levels of ROS, and since, in this study, HDF were exposed to two different oxygen tensions, it was important to investigate not only the levels of mitochondrial superoxide anion as reported above but also the levels of intracellular ROS generated under the two culture conditions. In order to quantify ROS production, the ROS index probe, carboxy-H_2_DCFDA, was employed. ROS production was calculated by measuring the percentage of cells showing an increase in green fluorescence. As reported in Figure 4(b), in increasing cell culture passages, there is a rise in intracellular ROS production which confirms the literature data and this is observed under both oxygen tensions. Surprisingly, in comparing the two culture conditions, a significantly higher percentage of HDF with high levels of ROS was observed under 5% O_2_ tension with respect to 21% O_2_ cultures in both young and old cells. This increased intracellular ROS production in HDF under 5% O_2_ appears to contrast with that observed in mitochondria reported in Figure 4(a) concerning superoxide anion radical production. Since ROS levels are known to affect telomere length, we also examined this parameter. The results indeed show that telomere length was greater in cells cultured under 21% O_2_ with lower levels of ROS than in HDF cultured under 5% O_2_ and that this was significant only for old cells (Supplementary Figure S1).

### 3.6. Total Coenzyme Q_10_ Levels Decrease with Cell Passages and Increase with Oxygen Tension

**Figure 5 fig5:**
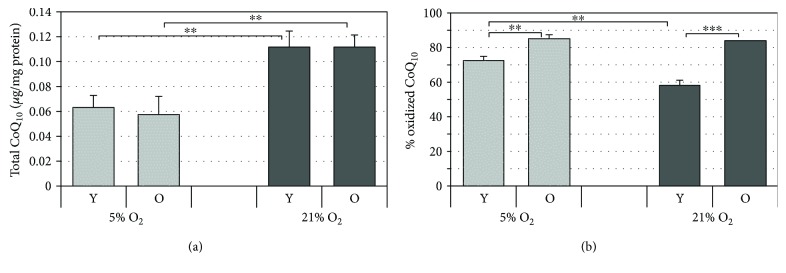
Levels of coenzyme Q_10_ in HDF analyzed by HPLC. HDF were serially passaged under the two oxygen tensions (21% and 5%), and at matched cell passage, intracellular CoQ_10_ was analyzed at various stages of cellular aging—young (Y) and old (O) HDF. Total CoQ_10_ levels (a) and % of oxidized CoQ_10_ (b) in HDF. Error bars represent ± SD. ^∗∗^ *p* < 0.01; ^∗∗∗^ *p* < 0.001.

CoQ_10_ is a key component in mitochondrial energy transfer, particularly in the electron transport chain, and it is endowed with notable antioxidant properties. During aging, the levels of this cofactor are known to decrease in different tissues including the skin [29], hence the relevance of its quantification to the aims of the present study. As reported in Figure 5(a), independently of the oxygen tension, comparing young and old cells, no significant differences were observed in terms of CoQ_10_ content while a significant increase in oxidized CoQ_10_ was observed during the aging process (Figure 5(b)). Moreover, cells cultured under 21% O_2_ have overall significantly higher levels of total CoQ_10_ compared to those cultured under 5% O_2_ at all cell passages tested. Furthermore, only young cells grown under 21% O_2_ presented lower levels of oxidized CoQ_10_ compared to those grown under 5% O_2_ while for old cells, the oxidized CoQ_10_ level was the same (Figure 5(b)).

Since many studies report that during serial passaging, the endogenous levels of CoQ_10_ decline especially in senescent cells, we evaluated total CoQ_10_ also in HDF senescent cells (>35th passage) under both oxygen tensions (Supplementary Figure S2). Indeed, we verified a depletion in total CoQ_10_ in senescent cells cultured under both oxygen tensions in agreement with the literature [29].

### 3.7. Total Glutathione Levels Reduce under Low Oxygen Tension

**Figure 6 fig6:**
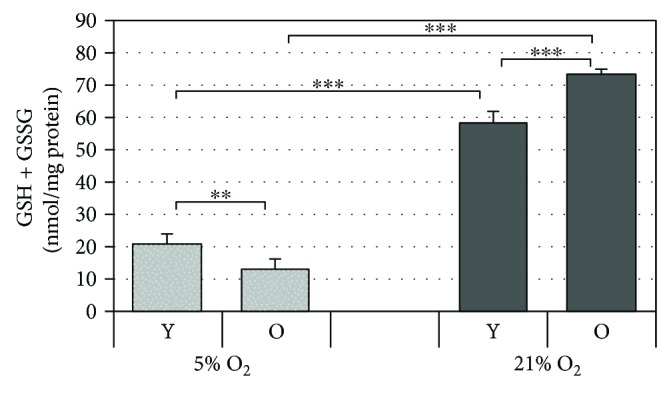
Levels of total intracellular glutathione analyzed in HDF using the glutathione reductase (GR) recycling assay. HDF were serially passaged under the two oxygen tensions (21% and 5%), and at matched cell passage, total glutathione (GSH + GSSG) was analyzed in young (Y) and old (O) HDF. Error bars represent ± SD. ^∗∗^ *p* < 0.01; ^∗∗∗^ *p* < 0.001.

Glutathione is an important nonenzymatic antioxidant that responds to the redox status of a cell by reacting directly with ROS [2, 30]; therefore, its levels were also evaluated in HDF under the two culture conditions. As shown in Figure 6, similarly to CoQ_10_, the concentration of the total glutathione measured was significantly higher in cells grown under 21% O_2_ with respect to that found in cells grown under 5% O_2_ in both young and old cells. Increasing levels of glutathione in cells exposed to a higher oxygen tension may be the result of adaptations due to an oxidant environment and could concur to lower cytosolic ROS as reported above.

### 3.8. Atmospheric Oxygen Tension Induces an Increase in SOD1 and SOD3 Gene Expression but Not in Catalase

**Figure 7:**
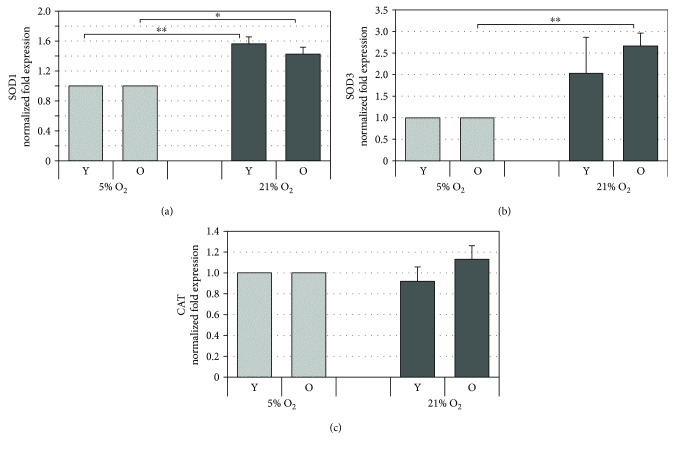
Gene expression analysis of superoxide dismutases (SOD) 1 and 3 and catalase (CAT), in HDF assessed by qPCR. The histograms show the modulation of SOD1 (a), SOD3 (b), and CAT (c) mRNA by oxygen tension (21% O_2_ vs 5% O_2_) at matched cell passages in young (Y) and old (O) HDF. The genes were normalized vs GAPDH/*β*-actin/SDHA and the results are reported as normalized fold expression. Error bars represent SD. ^∗^ *p* < 0.05; ^∗∗^ *p* < 0.01.

Part of the defense mechanism that has evolved to limit cellular damage caused by ROS is the repertoire of antioxidant enzymes that remove ROS before they can inflict damage. In order to determine whether this type of antioxidant response was modulated under our experimental conditions, the gene expression of some antioxidant enzymes was evaluated. These were cytoplasmic SOD1, extracellular SOD3, and catalase. The SOD enzymes detoxify superoxide anion radical by dismutating it to hydrogen peroxide, which is then removed by catalase. We compared by qPCR the gene expression of cytoplasmic SOD1, extracellular SOD3, and catalase using cells grown under 5% O_2_ tension as the control. As reported in Figure 7(a), SOD1 expression resulted upregulated under 21% O_2_ tension both in young and in old cells, while for SOD3, a statistically significant increase in its expression was observed only when comparing old cells (Figure 7(b)). However, higher SOD3 mRNA levels were also observed in young cells cultured under 21% O_2_ compared to 5% O_2_. Instead, no significant differences in mRNA levels were observed for catalase (Figure 7(c)).

### 3.9. Atmospheric Oxygen Tension Induces an Increase in MMP1 and a Decrease in COL1A1 Gene Expression

**Figure 8:**
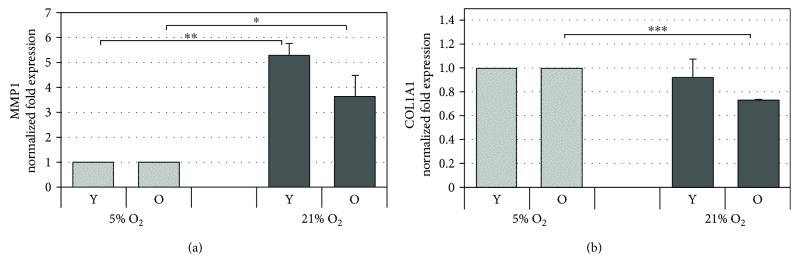
Gene expression analysis of matrix metalloproteinase 1 (MMP1) and *α*1(I)-procollagen (COL1A1) assessed by qPCR. The histograms show the modulation of the MMP1 (a) and COL1A1 (b) mRNAs by oxygen tension (21% O_2_ vs 5% O_2_) at matched cell passages in young (Y) and old (O) HDF. The genes were normalized vs GAPDH/*β*-actin/SDHA and the results are reported as normalized fold expression. Error bars represent SD. ^∗^ *p* < 0.05; ^∗∗^ *p* < 0.01; ^∗∗∗^ *p* < 0.001.

To examine the modulation of genes involved in extracellular matrix (ECM) remodeling by 21% O_2_ tension with respect to 5% O_2_ tension taken as control, we carried out a comparative gene expression analysis on COL1A1 and MMP1, two important skin-related genes that are modulated during skin aging responsible for the appearance of the prominent, characteristic phenotypic features of this process: wrinkles and sagging [31, 32]. Both these genes are targets for potential antiaging strategies. The results reported in Figure 8(a) show that there is a significant activation of MMP1 mRNA expression in both young and old cells, indicating that HDF cultured under 21% O_2_ are more aged with respect to those cultured under 5% O_2_. The same statement is valid also for mRNA expression of COL1A1 which is significantly downregulated under 21% O_2_ in old cells compared to 5% O_2_, an indication that cells are more aged (Figure 8(b)).

### 3.10. Atmospheric Oxygen Tension Leads to Increased DNA Damage in Older Cells

**Figure 9:**
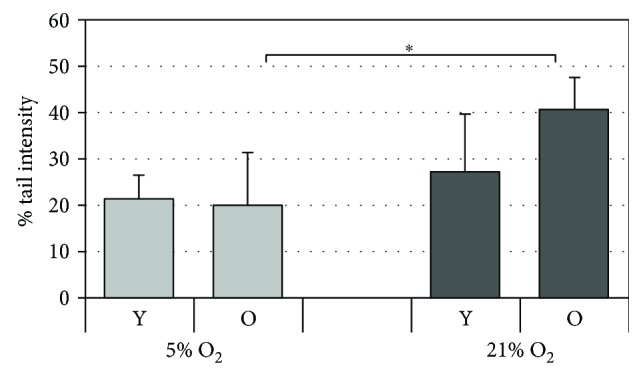
Cellular DNA damage in HDF assessed using the standard alkaline comet assay. HDF were serially passaged under the two oxygen tensions (21% O_2_ and 5% O_2_), and at matched cell passage, the intensity of fluorescence in the tail was analyzed in young (Y) and old (O) HDF. The data are reported as the average of the median values of tail intensity. Error bars represent ± SD. ^∗^ *p* < 0.05.

Aging cells are known to accumulate DNA damage and is a significant contributor to many age-related diseases [33]. To determine and compare the DNA integrity of HDF grown under the two different oxygen tensions, we employed the classical alkaline version of the comet assay which allows visualization of damaged DNA in individual cells.  Figure 9 shows that in cells cultured under 5% O_2_, DNA damage expressed as percentage of tail intensity fluorescence appears to be similar in both young and old cells. However, under 21% O_2_, a greater amount of DNA damage was observed in old cells, i.e., on increasing cell passages, compared to younger cells. This difference between the two culture conditions could be due to a better DNA repair system present at 5% O_2_, which is still capable of efficiently repairing any DNA damage that the cells experience even in the older cells. Moreover, the DNA analysis showed higher levels of damage in cells grown under 21% O_2_ compared to 5% O_2_, which was statistically significant only for cells at later passages (O).

## 4. Discussion

In this study, we examined the influence of oxygen tension on determining cellular aging in HDF and how this impacted ROS production, the enzymatic and nonenzymatic antioxidant response system, and the efficacy of this defense system in limiting DNA damage and in modulating gene expression of proteins involved in the ECM remodeling, linked to skin aging. For these purposes, we investigated a selection of parameters that represent and reflect the behavior of cellular responses to aging and oxygen tension.

First, cell aging due to serial passaging of HDF under both culture conditions was confirmed by all parameters that were considered (*β*-galactosidase activity, p16 expression, and proliferation rate). The results obtained are consistent with the theory of replicative cellular senescence whereby cells undergo a limited number of cumulative population doublings (CPD) before irreversible loss of proliferative capacity that is accompanied by several phenotypical, biochemical, and molecular alterations [19, 34]. This condition is most often described in the serial cultivation of fibroblasts typically used as a model of cell aging as we have used here [35]. Concerning with the oxygen tension of the two culture conditions, this indeed affects the aging process of serially cultured HDF. At matched cell passages, HDF are more aged under normoxic condition (21% O_2_) than under a hypoxic one (5% O_2_). This is particularly evident from the results obtained after the *β*-galactosidase and p16 gene expression assays. The increased SA-*β*-gal activity observed in senescent HDF can be attributed to increased lysosomal content which can be linked to increased autophagy that accompanies *in vitro* aging upon subculturing [36]. The fact that this activity was increased under atmospheric oxygen indicates that standard culture conditions induce “premature” aging in HDF. Likewise, the cyclin-dependent kinase inhibitor and tumor suppressor gene, p16, a hallmark of senescence [34], is increased dramatically in both young and old cells under 21% O_2_ with respect to 5% O_2_ cultures. This is in agreement with data in the literature where reduced expression of p16 was observed in human embryonic diploid fibroblasts grown under hypoxia (1.5% and 3% O_2_) [37]. Our results showed correlation between aging progress and proliferation index (Figure 1(c)). Indeed, the cells cultured under 5% O_2_ were divided a fewer number of times during the experimental course and also showed a slower rate of aging compared to the cells cultured under 21% O_2_.

Moreover, at the molecular level, in comparing HDF cultured under 21% with those at 5% O_2_, we observed a slight downregulation in miR-181a in young cells while the same microRNA resulted significantly upregulated in old cells. miR-181a has been shown to play a role in mitochondrial functionality, oxidative stress, and aging. In fact, previous studies have shown that transfection of dermal fibroblasts with miR-181a produced a senescent phenotype characterized by a decreased proliferation rate, increased positivity to *β*-gal, expression of p16, and decreased synthesis of COL16A1 [38]. In another study, on cultured astrocytes, inhibition of miR-181a improved mitochondrial functionality leading to a decrease in ROS and decreased mitochondrial susceptibility to depolarization [39]. Finally, Wang et al., using gain-of-function and loss-of-function approaches, verified that GPx-1 (glutathione peroxidase 1) expression in H9c2 cells was regulated by miR-181a in unstimulated cells. In particular, downregulation of miR-181a expression protected against the H_2_O_2_-induced injury of H9c2 cells by restoring cellular morphology and inhibiting ROS production [27].

Interestingly, the reduced levels of ROS observed correlate with the increased mRNA expression levels of the antioxidant enzymes, SOD3 and SOD1, and with the increased levels of the nonenzymatic antioxidants, coenzyme Q_10_ and glutathione, in HDF cultured under 21% O_2_ tension (Figures 4 [Fig fig5] [Fig fig6]–7). These findings appear to suggest that there is an adaptive biochemical response determined by oxygen tension levels. If ROS are the primary catalysts of oxygen toxicity, then antioxidant defenses should increase in response to increasing oxidant production resulting from the higher oxygen tension. In fact, it has been speculated that the generation of mitochondrial ROS has not been completely eliminated during evolution because they are involved in activating genes that help in the adaptation to stress, such as changes in oxygen levels [40]. Indeed, under 21% O_2_, we detected higher levels of mitochondrial superoxide anion radical compared to the cell cultures under hypoxia (Figure 4(a)). ROS are known to act as second messengers for signal transduction [41] resulting in the induction of detoxification enzymes via transcription factors. These include the antioxidant response element (ARE) binding proteins (e.g., members of the Nrf family), activator protein 1 (AP-1) and NF-*κ*B [42, 43].

In our experimental conditions, the increase in the antioxidant enzymes studied, with the exception of catalase, and the increase in total coenzyme Q_10_ and glutathione may hence account for the reduced intracellular ROS levels that we observe in cells grown under 21% O_2_ tension. These results are consistent with findings from other groups that show increased SOD activity in animal tissues and in some types of human cell cultures, including fibroblasts exposed to high oxygen tension [37, 44]. In our study, we did not observe significant changes in catalase, which suggests that it is probably not a significant factor in the removal of peroxides due to its low activity and relatively high K_m_ [45]. GSH peroxidases may be more important in eliminating endogenously produced hydrogen peroxide given that it also has a lower K_m_. Indeed, overexpression of GPx-1 was found to increase resistance to oxygen toxicity [46] which implicates requirement for GSH. GSH is reported to be consistently stimulated by increased oxygen tension in agreement with our findings and by endogenously and exogenously added active oxygen species, since this molecule is known to be responsive to the redox state of the cellular environment [47–49]. The mechanisms involved occur through transcriptional regulation of genes that regulate glutathione synthesis as well as uptake of GSH precursors most likely through the ARE element [50]. Notably, increase in miR-181a in old cells at 21% O_2_ may account for the inhibition of GPx-1, as shown previously, clarifying the loss of effective antioxidant responses in senescent cells.

There is no data in the literature regarding the role of oxygen tension on the endogenous levels of the bioenergetic and antioxidant molecule, coenzyme Q_10_ in HDF. However, from the data obtained with the SOD enzymes and with GSH, we infer that the signaling pathways triggered by increased superoxide levels in mitochondria may lead to stimulation of CoQ_10_ synthesis as an adaptive response [51]. In addition, besides the higher levels of total CoQ_10_, lower levels of oxidized CoQ_10_ were observed under 21% O_2_, a condition probably associated with an enhanced activity of CoQ_10_-reducing enzymes or lower levels of oxidizing species. The lower intracellular ROS levels that are concomitant with the increased antioxidant defense systems observed in our study under 21% O_2_ compared to 5% are in agreement with other literature reports that demonstrate increased ROS levels under hypoxia in cell cultures including fibroblasts [37, 40, 47]. Under low oxygen concentration, we also observed a slower proliferation rate compared to passage-matched cells cultured under a higher oxygen concentration, a finding that was also observed by Siddiqui et al. who showed that HDF grown chronically at 1% O_2_ proliferated at a rate 3 times slower than fibroblasts grown under standard culture conditions [52]. The greater antioxidant response observed under 21% O_2_ may also explain why we observed a higher proliferative capacity of HDF under this condition especially in young cells, since mild levels of oxidants are known to trigger proliferation [49, 53]. Interestingly, SOD3, which was significantly upregulated in our study in cells under 21% O_2_, has been shown to promote cell proliferation by binding to lipid rafts on cellular membranes, thereby influencing the activity of protein tyrosine phosphatases, downstream signal transduction pathway activation, and growth-related gene expression [54, 55]. This could in part account for the increased proliferation rate that we observed here.

In summarizing the above results, the cells cultured under 21% O_2_ are grown under standard culturing conditions that are not generally considered an acute stress one. However, these cells produce, during cellular metabolism, a mildly higher quantity of ROS with respect to the cells cultured under 5% O_2_. ROS, at low concentrations, act as signaling molecules that stimulate proliferation activity, and this response was observed only in young cells and not in old cells where the proliferation index under both conditions of oxygen tension was comparable (Figure 1(c)). This last data indicates that in our experiments, the two different oxygen conditions do not influence the capacity of cells to recover from (re)seeding. Furthermore, under our experimental conditions, we observe that both young and old cells produce more mitochondrial superoxide anion when grown under 21% O_2_ but only young cells are able to adapt by upregulating their antioxidant defenses and efficiently minimize cytosolic ROS production. Under these mild oxidative conditions in young cells, ROS are kept under control and exert a proliferative stimulus which results significantly greater than those in young cells cultured under 5% O_2_. On the contrary, in old cells, upregulation of antioxidant defenses (both enzymatic and nonenzymatic antioxidants) is not sufficient to balance the oxidative status.

The results discussed above represent a homeodynamic adaptation to mild oxidative stress which is in line with the concept of hormesis. This is a phenomenon by which a low dose of toxic stimulus induces resistance against a rising stress of similar nature by activating one or more stress response pathways and its consequent stimulation of repair/defense mechanisms [56]. This concept is effective to explain the reduction in age-related accumulation of molecular damage, and the underlying molecular mechanisms could be used to develop new antiaging strategies. However, in our case, despite the increase in both the enzymatic and nonenzymatic antioxidant responses observed under normoxia compared to hypoxia, in the long term, the mechanisms appeared insufficient for delaying the aging process. In fact, upregulation of antioxidant defenses represents only “one side of the coin” contributing to the steady-state of free radicals in cells and per se does not provide a guarantee of cellular protection, as we have observed in older cells where these adaptations were not sufficient to prevent cellular damage. Indeed, besides showing increased levels in the aging markers, chronic exposure of serially passaged HDF (>30p) to atmospheric levels of oxygen lead to depletion of both CoQ_10_ in senescent cells (Figure S1), impaired expression levels of the ECM matrix proteins, and extensive DNA damage at matched cell passages with respect to cultures grown under 5% O_2_ tension. Oxidative stress is known to cause damage to DNA which increases significantly in senescent cells, and the levels correlate with aging and age-related diseases [57]. The comet assay allows visualization of damaged DNA in individual cells, and the comparison between old cells at 21% O_2_ and 5% O_2_ clearly shows that there is an increase in the former group. This is consistent with the findings of Parrinello et al. who showed that mouse embryonic fibroblasts accumulated less DNA damage under 3% O_2_ than at 20% O_2_ and with those of Bell et al. who found less DNA damage in normal human lung fibroblasts at 3% O_2_ despite significantly higher levels of intracellular ROS than at 21% O_2_ [38, 58]. MMP1 expression is known to be upregulated by ROS and is a specific marker of skin aging since this enzyme that degrades collagen increases with age [31]. Concomitantly, the expression of COL1A1 in the skin decreases with age [32]. Because in our system, cells grown under normoxia appear more aged compared to those under hypoxia, it is reasonable to observe increased MMP1 and decreased COL1A1 mRNA expression levels. However, in the literature, there are several reports indicating that MMP1 expression is upregulated under severe hypoxia in HDF (2% O_2_) [59, 60], whereas for COL1A1, there is conflicting data: Yamanaka and Ishikawa [60], on a 3D culture of HDF, observed slightly increased mRNA expression under hypoxic conditions (2% O_2_) after 24 h which then decreased significantly at 72 h. Herrick et al. also found decreased COL1A1 mRNA during severe hypoxia both in a monolayer and in a collagen gel culture of HDF [61]. In contrast, Falanga et al. have repeatedly reported increased upregulation of this gene during hypoxia in the same cells after a maximum of 72 h of exposure [62]. Finally, Kan et al. found no modulatory effect posed by oxygen tension to which HDF were exposed [59]. The most likely explanation is that our study was performed on cells serially cultured under chronic hypoxia (24p) whereas the results obtained from the above reports were from cultures under acute and severe hypoxia (2% O_2_ only 72 h) which is the experimental condition most widely used compared to chronic exposure to hypoxia.

## 5. Conclusion

Overall, under the experimental conditions employed in our system, it is clear that atmospheric levels of oxygen tension (21%) impose a mild oxidative stress on dermal fibroblasts which accelerates the aging process in culture compared to lower, physiological levels of oxygen (5%) where the underlying level of oxidative stress is reduced. Since the responses that we observed are induced solely by the different oxygen tensions experienced by HDF during serial passaging, it is plausible to state that cells grown under normoxia undergo a “stress-induced premature senescence” when compared to their matched counterparts grown under hypoxia. Finally, our study shows an interesting protective effect in relation to adaptive responses triggered by mild oxidative conditions. Although the adaptive responses were not able to delay or prevent aging with cell passaging, the acute stimuli of oxidants might represent an interesting cue for the development of novel hormetic skin antiaging treatments. In this respect, the modulation of miR-181a to different oxygen tensions and its potential role in altering the expression of antioxidant genes could represent an important molecular event in skin aging that may be addressed as a target for antiaging strategies.

## Data Availability

All the data used to support the findings of this study are available from the corresponding author upon request.

## Supplementary Materials

Supplementary Materials The supplementary materials include Table S1 showing the gene primer sequence (genes of interest and housekeeping genes) used in this study with a brief description of the qPCR reactions, Figure S1 showing the effects of different oxygen tensions on telomere length in HDF at various stages of cellular aging with a description of the analysis and interpretation of the results, and Figure S2 showing the total levels of CoQ10 in HDF at various stages of cellular aging under the different oxygen tensions with a brief description of the assay conducted using HPLC.
