# Supplementary material for: Modulation of Oxidative Status by Normoxia and Hypoxia on Cultures of Human Dermal Fibroblasts: How Does It Affect Cell Aging?
Source: Oxid Med Cell Longev. 2018 Sep 23;2018:5469159. doi: 10.1155/2018/5469159 (PMC6199889; doi:10.1155/2018/5469159)
Supplement: Supplementary Materials — The supplementary materials include Table S1 showing the gene primer sequence (genes of interest and housekeeping genes) used in this study with a brief description of the qPCR reactions, Figure S1 showing the effects of different oxygen tensions on telomere length in HDF at various stages of cellular aging with a description of the analysis and interpretation of the results, and Figure S2 showing the total levels of CoQ10 in HDF at various stages of cellular aging under the different oxygen tensions with a brief description of the assay conducted using HPLC. [file 5469159.f1.docx]

**SUPPLEMENTARY MATERIAL**

**Table S1.** *Gene primers sequence used in the present study.*

*
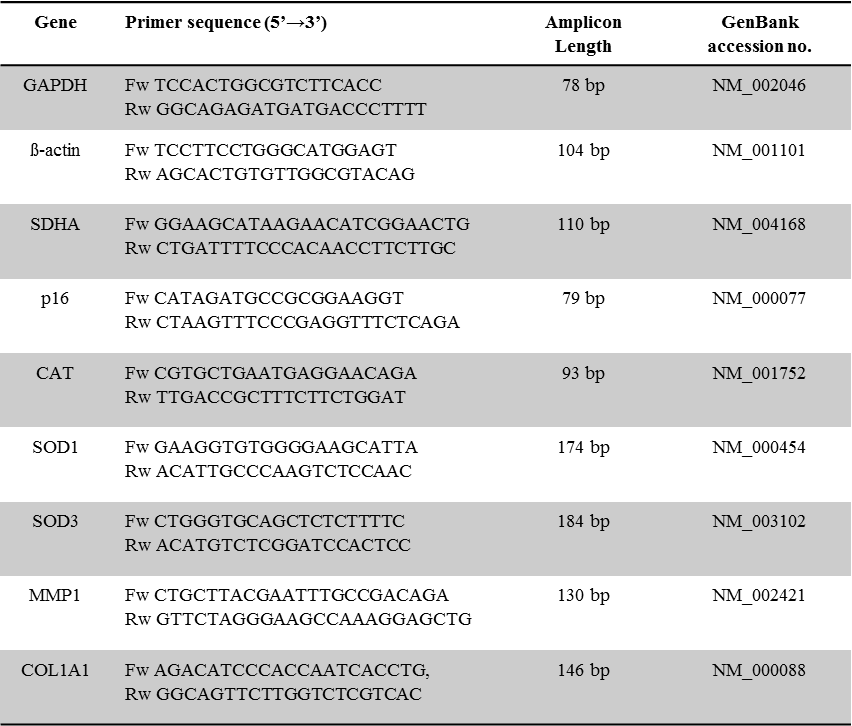
*

qPCR reactions were conducted on a MyiQ Single Color Real-Time PCR Detection System (Bio-Rad) in a 15 µL total reaction volume, using the iQ^TM^ SYBR Green Supermix (Bio-Rad). The primers sequences for the genes of interest: p16, CAT, SOD1, SOD3, MMP1 and COL1A1 are reported above (Tab. S1). All primers were used at a concentration of 400 nM, except for ß-actin that was used at a concentration of 300 nM. Each reaction was run in duplicate and for each gene a no-template control was included. The qPCR was programmed to start with a 3 min denaturation step at 95°C for polymerase activation, followed by 40 cycles of 15 sec denaturation at 95°C and 30 sec of annealing/extension at 60°C, during which fluorescence was measured. Next, a melting curve was constructed by increasing the temperature from 55 to 95°C in sequential steps of 0.5°C for 6 sec while continuously monitoring fluorescence. All PCR efficiencies were between 90 and 110%. For each sample at least three biological replicates were performed. The mRNA expression of the genes of interest was calculated according to the delta-delta Ct method (2^-ΔΔCt^) using the three reference genes GAPDH, β-actin and SDHA for normalization.

**Figure S1.** Telomere analysis in HDF cultured under the two oxygen tensions.


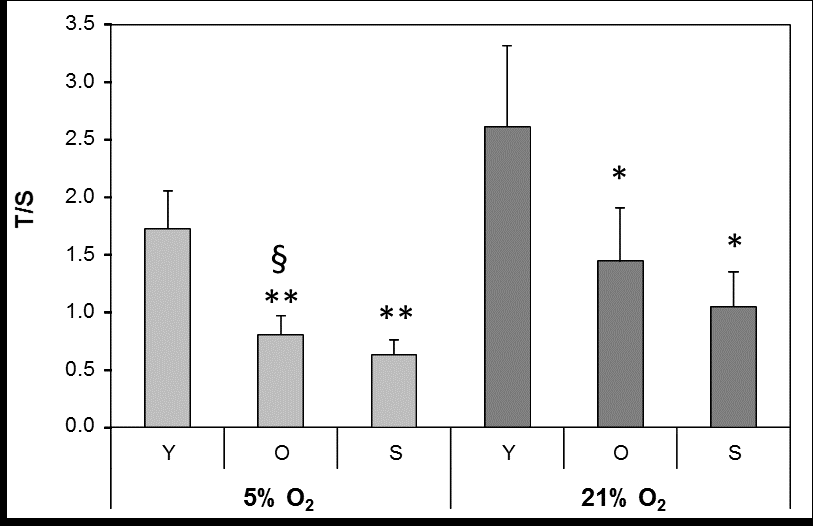


**Fig S1. Effects of oxygen tension on telomere length in HDF at various stages of cellular aging.** Telomere length was assessed using the relative telomere length analysis after qPCR and is reported as the T/S ratio in young (Y), old (O) and senescent (S) HDF. Error bars represent ± S.D. * p < 0.05 vs (Y) 21% O_2_, ** p < 0.01 vs (Y) 5% O_2_, § p < 0.05 vs (O) 21% O_2_.

*DNA extraction and telomere length analysis.* Total cellular DNA was isolated from the different passages using ExgeneTM Cell SV (GeneAll Biotechnology Co., LTD) according to the manufacturer’s instructions. Telomere length was assessed using the relative telomere length analysis (relative quantification is calculated by dividing the telomeric DNA product (T) by the reference gene (S), that is present as a single copy in the genome, to generate a T/S ratio) based on the quantitative polymerase chain reaction (PCR) which was carried out as described by Cawthon without any modifications (1). The primer sequences used for the telomeres (specifically designed by Cawthon for this assay) and for the single copy gene, 36B4 (acidic ribosomal phosphoprotein PO), are those reported by Cawthon. The results are reported as the T/S ratio.

Since telomere attrition is associated with senescence, telomere length was measured in HDF undergoing serial passaging under the two oxygen tensions. Telomere measurement was performed by quantitative PCR and is reported as telomere/single copy gene ratio (T/S). The results reported in Fig. 1S show a statistically significant shorter telomere length in old and senescent cells compared to younger fibroblasts both under standard culture conditions (21% O_2_) and under hypoxia (5% O_2_) consistent with literature findings (2). However, telomeres were longer in fibroblasts grown under atmospheric oxygen tension than in cells grown under hypoxia during all passages considered. However, it was only significant for old cells between 20-24p (O). This finding is consistent with previous data on telomere length in IMR90 human foetal lung fibroblasts grown in 20% O_2_ compared to 3% O_2_ (3).

We expected to observe shorter telomeres under atmospheric oxygen since telomeres are known to significantly shorten under mild oxidative stress and that ROS can affect telomere maintenance at multiple levels, even indirectly through their interaction with the catalytic subunit of telomerase in telomerase reverse transcriptase (TERT) (4). Serra et al. noted that SOD3 overexpression in fibroblasts decreases the peroxide content and the rate of telomere shortening (5) while others report that telomere shortening is largely dependent on the interplay of oxidative stress and antioxidant defence rather than the cell divisions (6). The reason why we found longer telomeres under a more oxidant condition (21% O_2_) may be related to the reduced levels of intracellular ROS found compared to those detected in HDF cultured under hypoxia at matched cell passages.

1. R.M. Cawthon, Telomere measurement by quantitative PCR, Nucleic Acids Res 30(10) (2002) e47.

(2) C.B. Harley, A.B. Futcher, C.W. Greider, Telomeres shorten during ageing of human fibroblasts, Nature 345(6274) (1990) 458-60.

(3) B. Britt-Compton, F. Wyllie, J. Rowson, R. Capper, E.J. R, M.B. D, Telomere dynamics during replicative senescence are not directly modulated by conditions of oxidative stress in IMR90 fibroblast cells, Biogerontology 10(6) (2009) 683-93

(4) T. Richter, T. von Zglinicki, A continuous correlation between oxidative stress and telomere shortening in fibroblasts, Exp Gerontol 42(11) (2007) 1039-42.

(5) V. Serra, T. von Zglinicki, M. Lorenz, G. Saretzki, Extracellular superoxide dismutase is a major antioxidant in human fibroblasts and slows telomere shortening, J Biol Chem 278(9) (2003) 6824-30.

(6) T. von Zglinicki, A. Burkle, T.B. Kirkwood, Stress, DNA damage and ageing -- an integrative approach, Exp Gerontol 36(7) (2001) 1049-62.

**Figure S2.** *Total levels of coenzyme Q_10_ in HDF analyzed by HPLC.*


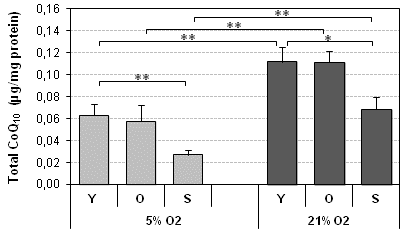


HDF were serially passaged under the two oxygen tensions (21% and 5%), and at matched cell passage intracellular CoQ_10_ content was analyzed at various stages of cellular aging; young (Y), old (O) and senescent (S) HDF. Error bars represent ± SD.* p<0.05; ** p<0.01; *** p<0.001.

Coenzyme Q_10_ (CoQ_10_) levels and its oxidative status were assayed in the fibroblasts grown on 6-well plates using a dedicated high-performance liquid chromatography (HPLC) system with electrochemical detector capable of detecting both reduced and oxidized forms (ECD; Shiseido, Tokyo, Japan) as reported in (1). CoQ_10_ concentration was verified by using a single dilution step. Briefly, after cell harvesting the cell pellet was resuspended in 50 μL PBS and extracted with 250 μL propanol. After vigorous vortexing, the extraction mixture was centrifuged for 1 min at 13,000*g*, 4°C and 40 μL of supernatant was injected into the HPLC system. Intracellular total CoQ_10_ levels are expressed as μg/mg of protein. The protein concentration was determined using the Bradford assay.

1. F. Olivieri, R. Lazzarini, L. Babini, F. Prattichizzo, M.R. Rippo, L. Tiano, S. Di Nuzzo, L. Graciotti, R. Festa, F. Bruge, P. Orlando, S. Silvestri, M. Capri, L. Palma, M. Magnani, C. Franceschi, G.P. Littarru, A.D. Procopio. Anti-inflammatory effect of ubiquinol-10 on young and senescent endothelial cells via miR-146a modulation. Free Radic Biol Med 63 (2013) 410-20.
